# Pericardial disease as a rare complication of pediatric appendicitis: a systematic literature search

**DOI:** 10.1186/s40981-020-00395-8

**Published:** 2020-11-09

**Authors:** Bibek Saha, Kazuyoshi Aoyama, Maria-Alexandra Petre, Marina Englesakis, James Robertson, Mark Levine

**Affiliations:** 1 John A. Burns School of Medicine, University of Hawaii at Manoa, 651 Ilalo St, Honolulu, HI 96813 USA; 2 Department of Anesthesia and Pain Medicine, The Hospital for Sick Children, 555 University Ave, #2211, Toronto, ON M5G 1X8 Canada; 3 Program in Clinical Health Evaluative Sciences, The SickKids Research Institute, 555 University Ave, #2211, Toronto, ON M5G 1X8 Canada; 4 Department of Pediatric Anesthesia, Montreal Children’s Hospital, 1001 Decarie Blvd, Montreal, QC H4A 3J1 Canada; 5 Library and Information Services, University Health Network, 200 Elizabeth St, Toronto, ON M5G 2C4 Canada

**Keywords:** Pericarditis, Pericardial effusion, Cardiac tamponade, Acute appendicitis, Pediatric anesthesia

## Abstract

**Background:**

Classic symptoms of acute appendicitis are well known but are uncommon and often misinterpreted in pediatric patients, potentially delaying diagnosis and resulting in rare sequelae.

**Methods:**

We conducted a comprehensive systematic literature search of case reports detailing pericardial disease as a rare complication of pediatric appendicitis through MEDLINE, Embase, and Cochrane Databases. Inclusion criteria was that the patient must be < 18 years old and present with both pericardial disease and appendicitis.

**Results:**

Our search yielded 7 cases with an average age of 10.3 ± 3.9 years old. The cases involved cardiac tamponade, pericarditis, and/or pericardial effusion. Five cases were diagnosed with appendicitis before complicated by pericardial disease. Most cases had an infectious component, but a majority had negative pericardial fluid cultures. Pleural effusion and abdominal abscesses were other common complications of pediatric appendicitis.

**Conclusion:**

Awareness of this uncommon relationship may have prognostic value as this may facilitate appropriate management of pericardial effusions, tamponade, and/or appendicitis.

**Supplementary Information:**

The online version contains supplementary material available at 10.1186/s40981-020-00395-8.

## Background

Acute appendicitis is the most common pediatric general surgical emergency [1]. Although the classic symptoms such as periumbilical pain migrating to the lower right quadrant and nausea are well known, they occur in less than 50% of children [2]. Additionally, many children without appendicitis present with these classic symptoms [2]. Furthermore, young children may not understand or be able to communicate these symptoms [2]. Taken together, making a diagnosis of acute appendicitis in the pediatric population may be challenging and/or delayed increasing the likelihood of perforation culminating in the development of rare complications [2, 3].

Pericardial disease, while uncommon, can be a life-threatening condition in the pediatric emergency department and must be identified and treated rapidly to prevent a poor outcome [4]. Previously, we reported of a unique case with cardiac tamponade complicating perforated appendicitis in a 7-year-old girl [5] prompting us to investigate the relationship between pericardial disease and pediatric appendicitis further. The main objective of the current study was to explore temporal nature of this relationship. Additionally, given that pericardial disease could cause significant hemodynamic instability during general anesthesia, we also aimed to summarize anesthetic management of the eligible cases. Here, we present a comprehensive systematic literature search of case reports detailing pericardial disease as a rare complication of appendicitis in the pediatric population and suggestions regarding management.

## Methods

An information specialist (M.E.) was enlisted to conduct an extensive systematic search through MEDLINE, MEDLINE In-Process/ePubs, Embase, Cochrane Database of Systematic Reviews, and Cochrane CENTRAL, starting from their inception. There were no language restrictions. Search strategies (Supplementary online resource 1) were built to contain sets of terms reflecting our topic of interest including pericardial disease (pericarditis, pericardial effusion, cardiac tamponade), appendicitis, and the patient population (pediatric patients). De-duplication and screening of articles were undertaken using reference management software DistillerSR. Specifically, two of the authors (B.S. and M.A.P.) independently screened all titles and abstracts retrieved in the literature search for relevancy. The remaining articles underwent full-text assessment by the reviewers to determine eligibility based on the inclusion criteria that the patient must be < 18 years old and present with both pericardial disease and appendicitis. Any disagreements between the two reviewers were resolved through discussion. Information regarding study ID (author information, year and country of publication), population demographics (age, gender), patient presentation (evidence of appendicitis, evidence of pericardial disease, evidence of infection or inflammation), associated complications (pleural effusions, abdominal abscesses), and patient management were collected into a standardized data extraction form.

## Results

**Fig. 1:**
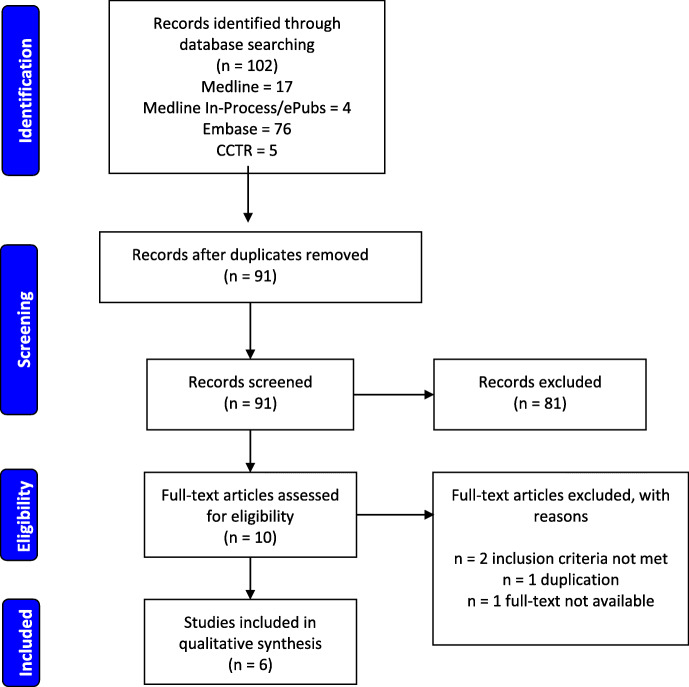
PRISMA flow diagram for search and review strategy

Our search yielded 102 titles but only 10 articles were eligible for full-text assessment after de-duplication and the screening procedure (Fig. 1). Three of these articles did not meet the inclusion criteria and a fourth article did meet the inclusion criteria based on the abstract but was not accessible for full-text assessment [6]. The remaining 6 articles [5, 7–11], including our own, encompassed 7 relevant case reports which were used for data synthesis. The cases span across more than a century (119 years) and took place in 6 different countries (Australia, Britain, Canada, Netherlands, Taiwan, and USA; Table 1). The majority of the patients were female (5/7) and the average age at presentation was 10.3 ± 3.9 years old with a range of 3–14 years (Table 1).

**Table 1:** Summary characteristics of case reports included in systematic literature search

Author	Year published	Country of publication	Age	Sex (M/F)	Description of appendicitis	Description of pericardial disease	Temporal relationship	Microbial analysis	Other parameters of infection/inflammation/SIRS	Pleural effusion present (Y/N)	Abdominal abscess present (Y/N)	Anesthetic management
Saha et al. [5]	2020	Canada	7	F	Acute suppurative appendicitis with perforation was diagnosed based on H & P, ultrasound, and later pathological analysis	Cardiac tamponade was diagnosed based on H & P, imaging, and EKG.	Symptoms related to appendicitis began 3 days before tamponade diagnosis. Appendicitis was diagnosed 3 days later.	The bloody sanguineous pericardial fluid tested negative for viral (CMV, EBV, HHV6, adenovirus), bacterial, and fungal infection. The serous pleural fluid was negative for bacterial culture. The blood, cerebrospinal fluid and urine also tested negative for viral, bacterial and fungal infection.	Elevated CRP; fever; tachycardia; tachypnea elevated WBC count (15.0 × 10^9^/L)	Y (right side)	Y (two interconnected abdominal abscesses)	Pericardiocentesis was performed under conscious sedation (ketamine, midazolam, morphine, and local anesthesia) while maintaining spontaneous ventilation. Afterwards, laparoscopic appendectomy was performed under general anesthesia (propofol, midazolam, fentanyl, rocuronium, sevoflurane, and morphine)
Ku et al. [8]	2017	Australia	14	M	Perforated appendicitis was diagnosed based on H & P.	Pericardial effusion was diagnosed based on H & P and imaging	Pericardial effusion was diagnosed at least 8 days after the diagnosis of appendicitis.	Microscopic analysis of the hemoserous pericardial fluid revealed gram-negative rods, gram-positive cocci, and gram-positive rods. The culture of the effusion grew enteric gram-negative rods and mixed anaerobes including *Streptococcus anginosus* (*S. milleri*).	Fever; tachypnea; other parameters not reported	Y (bilateral)	Y (multiple abdominal abscesses)	Not reported
Tan et al. [9]	2004	Netherlands	12	F	Severe periappendicitis was diagnosed based on H & P and pathologic examination after appendectomy	Pneumo-hydropericardium, recurrent pericardial effusions and constrictive pericarditis was diagnosed based on H & P and imaging.	Symptoms related to appendicitis began approximately 12 days before pericardial effusion was diagnosed. Appendicitis was diagnosed 16 days later. Nine days later she developed pericarditis.	Blood cultures were positive for *B. fragilis* and *S. milleri*; Culture of the serosanguinolent pericardial fluid was positive for *E. coli*, *S. viridans*, *C. albicans*, but no anaerobes; abdominal cultures grew aerobic gram-negative rods, anaerobic gram-positive cocci, and enterococci	Elevated ESR (41 mm/h); fever; tachypnea; elevated WBC count (27.1 × 10^9^/L); other parameters not reported	Y	Y (psoas abscess); lung abscess also present	Not reported
			13	F	Appendicitis was diagnosed based on H & P and during appendectomy, the appendix perforated. A fecalith was found in the appendix.	Purulent pericarditis was diagnosed based on H & P, imaging and EKG.	Appendicitis was diagnosed within 1–2 days of symptom presentation. Pericarditis was diagnosed after at least 17 days.	Pus evacuation occurred through the vagina and culture of the specimen grew *E. coli*, anaerobic rods (*B. vulgatus*), and peptostreptococcus species. Cultures of the pericardial fluid were negative.	Normal ESR (17 mm); fever; normal WBC count (11.5 × 10^9^/L); Other parameters not reported	Not reported	Y (multiple intraabdominal abscesses including a Douglas abscess and subphrenic abscess)	Not reported
Kao et al. [7]	2002	Taiwan	3	F	Ruptured retrocecal appendix with an appendicolith was diagnosed based on H & P	Pericardial effusion diagnosed based on H & P	Symptoms related to appendicitis began at least 7 days prior to the diagnosis of a ruptured retrocecal appendix. Pericardial effusion was diagnosed at least 4 days later.	Culture of the abscess yielded group D beta-hemolytic streptococcus, *E. coli*, *B. ovatus*, and *B. fragilis*. The culture of the pleural fluid grew *E. coli* and *B. ovatus*. Urine, blood, and pericardial fluid cultures were all negative.	Fever; tachypnea; elevated WBC count (32,810/mm^3^) with left shift; other parameters not reported	Y (right side) and empyema	Y (right perinephric abscess)	Not reported
Speirs [11]	1951	Britain	11	M	Gangrenous appendix with pus in the peritoneal cavity was diagnosed based on H & P	Recurrent pericardial effusions with pericarditis diagnosed based on H & P and imaging.	Appendicitis was diagnosed within 2 days of symptom presentation. Recurrent pericarditis with pericardial effusion was diagnosed months later.	Culture of liver abscesses grew coliform organisms, non-hemolytic streptococci and *S. albus*. The pericardial fluid which was greenish-yellow and opalescent contained polymorphonuclear leukocytes and lymphocytes but was sterile when cultured	Fever; other parameters not reported	Y (right side)	Y (including liver abscesses)	Not reported
Mann [10]	1901	USA	12	F	Diagnosis of appendicitis was made based on H & P	Diagnosis of pericardial effusion and suppurative pericarditis was made based on H & P and gross examination	Appendicitis was diagnosed within 3–4 days after symptom presentation. Pericarditis with pericardial effusion was diagnosed 8–9 days later.	Septic shock; the bloody purulent pericardial fluid was positive for abundant pneumococcus	Fever; tachycardia; tachypnea; other parameters not reported	Not reported	Not reported	Ether was used for the pericardiocentesis; other information not reported

*H & P* history and physical, *EKG* electrocardiogram, *CMV* cytomegalovirus, *EBV* Epstein-Barr virus, *HHV6* Human Herpesvirus 6, *WBC* white blood cell, *ESR* erythrocyte sedimentation rate, *CRP* C-reactive protein, *SIRS* systemic inflammatory response syndrome

Two cases presented with pericardial effusion alone, 1 case presented with pericarditis alone, 3 cases presented with both pericarditis and pericardial effusion, and 1 case presented with cardiac tamponade secondary to appendicitis (Table 1). In the majority of cases (5/7), appendicitis was diagnosed before pericardial disease, but in the remaining cases, this order was reversed (Table 1). Only two out of six articles described anesthetic management of a child with a pericardial disease in the context of appendicitis [5, 10]. The one published in 1901 described the use of Ether for their management but did not discuss potential hemodynamic instability of the case. Thus, our own case was the only report that sufficiently described such anesthetic management from a hemodynamic perspective, which underscored the maintenance of spontaneous ventilation with conscious sedation for the emergent pericardiocentesis prior to appendectomy (Table 1) [5].

All but one of the cases had some sort of infectious component (Table 1). However, cultures of the pericardial fluid were negative for 4/7 cases (Table 1). All patients were febrile during their hospital stay, where 3/7 patients had an elevated white blood cell (WBC) count, 1 patient had an elevated c-reactive protein (CRP) and another patient had an elevated erythrocyte sedimentation rate (ESR; Table 1). The criteria for systemic inflammatory response syndrome (SIRS) was met in 5/7 cases with the remaining cases lacking information to make an assessment. Pleural effusion, which was predominantly right sided or bilateral, complicated 5/7 cases (Table 1). Almost all cases (6/7) were complicated by one or multiple abdominal abscesses (Table 1).

## Discussion

Although acute appendicitis is the major cause of emergency surgery in the pediatric population, diagnosing this condition in children remains challenging due to the fact that symptoms are not always typical and are often mistaken for gastroenteritis [2, 3]. A delayed diagnosis can lead to an increased risk of complications and associated morbidity and mortality [3, 8]. Cardiac tamponade is a life-threatening condition that is caused by cardiac compression secondary to fluid or gas accumulation in the pericardial space [4]. Major causes of tamponade include pericardial effusion, chest trauma, cardiac wall rupture, and aortic dissection. Additionally, pericardial effusion can be idiopathic or secondary to pericarditis, malignancy, uremia, infection, radiation, post-acute myocardial infarction, autoimmune disorders, collagen vascular disease, and hypothyroidism [4, 8].

To our surprise, the first reported case that detailed the rare relationship between pericardial disease and appendicitis in the pediatric population occurred in 1901 and 5/7 cases were reported after 2000 (Table 1). Additionally, the vast majority of cases were reported in developed countries (Table 1). Taken together, this may suggest that pericardial disease may be a more common complication of pediatric appendicitis but was under reported in the 1900s and in developing countries. Although in most of the cases, appendicitis was diagnosed prior to pericardial disease, which is in accordance with the idea that pericardial disease develops as a complication of appendicitis, in 2/7 cases, pericardial disease was diagnosed before appendicitis. Therefore, in the presence of pericardial disease, especially with symptoms associated with appendicitis, a workup of appendicitis may be warranted. Pleural effusions and abdominal abscesses were the other common complications of the appendicitis (Table 1).

The main finding of the current study was that we were able to characterize the association between pediatric appendicitis and pericardial disease. However, one limitation of our study is that we were unable to propose a definitive mechanism that explains the connection between these two conditions. Nevertheless, here, we describe a few possible mechanisms. First, the pericardium may have simply been directly infected due to bacteremia secondary to appendicitis. However, a majority of the cases we presented herein had negative cultures of the pericardial fluid (Table 1). While we were unable to assess 2/7 cases for the presence of SIRS due to a lack of information, the remaining cases all met the diagnostic criteria (Table 1). Therefore, SIRS secondary to the appendicitis may have been contributory to the pericardial disease. Similarly, in our previous case report, we attributed SIRS secondary to perforated appendicitis as the cause of the cardiac tamponade after ruling out/providing evidence against other likely etiologies which included autoimmune/rheumatologic diseases, malignancy, infection, and trauma [5]. Additionally, another mechanism contributing to the etiology of pericardial disease may have been the contiguous spread of inflammation and/or infection from the retroperitoneal space to the mediastinum [12–14]. Finally, 71% of the cases were females (Table 1), and this may have been because women are more commonly affected by systemic inflammatory diseases (SID) than men and are more commonly affected by pericarditis related to SID [15].

With regard to our secondary objective to summarize anesthetic management of a child with a pericardial disease in the context of appendicitis, all cases, but our own, did not report the anesthetic management from a hemodynamic point of view (Table 1). Therefore, given the lack of data, we were unable to summarize such anesthetic management.

In conclusion, diagnosis of acute appendicitis may be difficult and, therefore, delayed in the pediatric population resulting in a perforated appendix and associated rare complications. We should consider the presence of life-threatening pericardial disease when anesthetizing children with appendicitis. Pericardial diseases associated with pediatric appendicitis may be due to direct infection, SIRS, or the contiguous spread from retroperitoneal space to the mediastinum. Awareness of this uncommon association may have prognostic value as this may facilitate appropriate management of pericardial effusions, tamponade, and/or appendicitis.

## Supplementary Information

**Additional file 1.** Summary of Search Strategies.

## Data Availability

All available data and materials are presented in the main and supplemental documents.

## Abbreviations

CRP: C-reactive protein
ESR: Erythrocyte sedimentation eate
WBC: White blood cells
