# Supplementary material for: Pericardial disease as a rare complication of pediatric appendicitis: a systematic literature search
Source: JA Clin Rep. 2020 Nov 9;6:89. doi: 10.1186/s40981-020-00395-8 (PMC7652975; doi:10.1186/s40981-020-00395-8)
Supplement: Supplementary file 1 — Additional file 1. Summary of Search Strategies. [file 40981_2020_395_MOESM1_ESM.docx]

**Supplementary online resource 1:** Summary of Search Strategies

# Medline

Ovid MEDLINE(R) 1946 to January 02, 2020

| **#** | **Searches** | **Results** |
| --- | --- | --- |
| 1 | Cardiac Tamponade/ | 6230 |
| 2 | (cardia? adj2 tamponad*).mp. | 8082 |
| 3 | (pericardia* adj2 tamponad*).mp. | 1379 |
| 4 | pericardial effusion/ | 8447 |
| 5 | (pericardial adj2 effus*).mp. | 12270 |
| 6 | (peri-cardial adj2 effus*).mp. | 2 |
| 7 | (pericardi* adj2 tamponad*).mp. | 1636 |
| 8 | (peri-cardi* adj2 tamponad*).mp. | 0 |
| 9 | (heart? adj2 tamponad*).mp. | 104 |
| 10 | (myocardia* adj2 tamponad*).mp. | 54 |
| 11 | chylopericardi???.mp. | 309 |
| 12 | haemopericardi???.mp. | 172 |
| 13 | hemopericardi???.mp. | 680 |
| 14 | ((compress* adj3 heart?) and (increas* adj3 intrapericardial pressure?)).mp. | 2 |
| 15 | ((compress* adj3 heart?) and (increas* adj3 intra-pericardial pressure?)).mp. | 1 |
| 16 | pericarditis/ | 8611 |
| 17 | pericarditis.mp. | 14201 |
| 18 | or/1-17 [ Cardiac Tamponade & related terms ] | 29252 |
| 19 | Appendicitis/ | 18797 |
| 20 | Appendix/ | 6733 |
| 21 | appendectomy/ | 10838 |
| 22 | appendicit*.mp. | 21603 |
| 23 | appendix??.mp. | 15371 |
| 24 | appendices.mp. | 1426 |
| 25 | appendiceal*.mp. | 5627 |
| 26 | appendicular*.mp. | 4655 |
| 27 | appendicolith*.mp. | 207 |
| 28 | appendicectom*.mp. | 1973 |
| 29 | appendectom*.mp. | 13300 |
| 30 | appendicopath*.mp. | 59 |
| 31 | caecal appendage?.mp. | 2 |
| 32 | cecal appendage?.mp. | 2 |
| 33 | vermiform process??.mp. | 103 |
| 34 | vermix.mp. | 16 |
| 35 | or/19-34 [ related to Appendix ] | 41881 |
| 36 | 18 and 35 [ Cardiac Tamponade + Appendix ] | 33 |
| 37 | limit 36 to ("all adult (19 plus years)" or "young adult (19 to 24 years)" or "adult (19 to 44 years)" or "young adult and adult (19-24 and 19-44)" or "middle age (45 to 64 years)" or "middle aged (45 plus years)" or "all aged (65 and over)" or "aged (80 and over)") | 20 |
| 38 | 36 not 37 | 13 |
| 39 | limit 36 to ("all infant (birth to 23 months)" or "all child (0 to 18 years)" or "newborn infant (birth to 1 month)" or "infant (1 to 23 months)" or "preschool child (2 to 5 years)" or "child (6 to 12 years)" or "adolescent (13 to 18 years)") | 12 |
| 40 | 38 or 39 | 17 |
| 41 | remove duplicates from 40 | 17 |

# Medline In-Process

Ovid MEDLINE(R) Epub Ahead of Print and In-Process & Other Non-Indexed Citations January 02, 2020

| **#** | **Searches** | **Results** |
| --- | --- | --- |
| 1 | Cardiac Tamponade/ | 0 |
| 2 | (cardia? adj2 tamponad*).mp. | 746 |
| 3 | (pericardia* adj2 tamponad*).mp. | 177 |
| 4 | pericardial effusion/ | 0 |
| 5 | (pericardial adj2 effus*).mp. | 1156 |
| 6 | (peri-cardial adj2 effus*).mp. | 0 |
| 7 | (pericardi* adj2 tamponad*).mp. | 213 |
| 8 | (peri-cardi* adj2 tamponad*).mp. | 0 |
| 9 | (heart? adj2 tamponad*).mp. | 5 |
| 10 | (myocardia* adj2 tamponad*).mp. | 7 |
| 11 | chylopericardi???.mp. | 26 |
| 12 | haemopericardi???.mp. | 19 |
| 13 | hemopericardi???.mp. | 85 |
| 14 | ((compress* adj3 heart?) and (increas* adj3 intrapericardial pressure?)).mp. | 0 |
| 15 | ((compress* adj3 heart?) and (increas* adj3 intra-pericardial pressure?)).mp. | 0 |
| 16 | pericarditis/ | 0 |
| 17 | pericarditis.mp. | 1034 |
| 18 | or/1-17 [ Cardiac Tamponade & related terms ] | 2592 |
| 19 | Appendicitis/ | 0 |
| 20 | Appendix/ | 0 |
| 21 | appendectomy/ | 0 |
| 22 | appendicit*.mp. | 2592 |
| 23 | appendix??.mp. | 2553 |
| 24 | appendices.mp. | 211 |
| 25 | appendiceal*.mp. | 749 |
| 26 | appendicular*.mp. | 857 |
| 27 | appendicolith*.mp. | 49 |
| 28 | appendicectom*.mp. | 334 |
| 29 | appendectom*.mp. | 1252 |
| 30 | appendicopath*.mp. | 2 |
| 31 | caecal appendage?.mp. | 0 |
| 32 | cecal appendage?.mp. | 0 |
| 33 | vermiform process??.mp. | 1 |
| 34 | vermix.mp. | 2 |
| 35 | or/19-34 [ related to Appendix ] | 5989 |
| 36 | 18 and 35 [ Cardiac Tamponade + Appendix ] | 4 |

# Embase

Embase Classic+Embase 1947 to 2020 January 02

| **#** | **Searches** | **Results** |
| --- | --- | --- |
| 1 | Cardiac Tamponade/ | 9477 |
| 2 | heart tamponade/ [ Embase ] | 16118 |
| 3 | (cardia? adj2 tamponad*).mp. | 9187 |
| 4 | (pericardia* adj2 tamponad*).mp. | 2547 |
| 5 | pericardial effusion/ | 25933 |
| 6 | (pericardial adj2 effus*).mp. | 29312 |
| 7 | (peri-cardial adj2 effus*).mp. | 17 |
| 8 | (pericardi* adj2 tamponad*).mp. | 3050 |
| 9 | (peri-cardi* adj2 tamponad*).mp. | 1 |
| 10 | (heart? adj2 tamponad*).mp. | 16166 |
| 11 | (myocardia* adj2 tamponad*).mp. | 101 |
| 12 | chylopericardi???.mp. | 471 |
| 13 | haemopericardi???.mp. | 394 |
| 14 | hemopericardi???.mp. | 2529 |
| 15 | ((compress* adj3 heart?) and (increas* adj3 intrapericardial pressure?)).mp. | 3 |
| 16 | ((compress* adj3 heart?) and (increas* adj3 intra-pericardial pressure?)).mp. | 2 |
| 17 | pericarditis/ | 18182 |
| 18 | pericarditis.mp. | 25139 |
| 19 | or/1-18 [ Cardiac Tamponade & related terms ] | 61344 |
| 20 | Appendicitis/ | 21345 |
| 21 | Appendix/ | 6766 |
| 22 | appendectomy/ | 21823 |
| 23 | exp appendix disease/ [ Embase ] | 34461 |
| 24 | appendicit*.mp. | 32782 |
| 25 | appendix??.mp. | 28076 |
| 26 | appendices.mp. | 2481 |
| 27 | appendiceal*.mp. | 6593 |
| 28 | appendicular*.mp. | 8212 |
| 29 | appendicolith*.mp. | 353 |
| 30 | appendicectom*.mp. | 3661 |
| 31 | appendectom*.mp. | 23830 |
| 32 | appendicopath*.mp. | 98 |
| 33 | caecal appendage?.mp. | 2 |
| 34 | cecal appendage?.mp. | 3 |
| 35 | vermiform process??.mp. | 118 |
| 36 | vermix.mp. | 33 |
| 37 | or/20-36 [ related to Appendix ] | 69636 |
| 38 | 19 and 37 [ Cardiac Tamponade + Appendix ] | 150 |
| 39 | limit 38 to (conference abstracts or (conference abstract or "conference review") or (book or book series)) | 22 |
| 40 | 38 not 39 | 128 |
| 41 | (exp animals/ or exp animal experimentation/ or nonhuman/) not ((exp animals/ or exp animal experimentation/ or nonhuman/) and exp human/) | 7161682 |
| 42 | 40 not 41 | 124 |
| 43 | limit 40 to human | 108 |
| 44 | 42 or 43 | 124 |
| 45 | limit 44 to (adult <18 to 64 years> or aged <65+ years>) | 56 |
| 46 | 44 not 45 | 68 |
| 47 | limit 44 to (embryo <first trimester> or infant <to one year> or child <unspecified age> or preschool child <1 to 6 years> or school child <7 to 12 years> or adolescent <13 to 17 years>) | 28 |
| 48 | 46 or 47 | 77 |
| 49 | remove duplicates from 48 | 76 |

# CCTR

Cochrane Central Register of Controlled Trials 2014 to Present

| **#** | **Searches** | **Results** |
| --- | --- | --- |
| 1 | Cardiac Tamponade/ | 24 |
| 2 | heart tamponade/ [ Embase ] | 0 |
| 3 | (cardia? adj2 tamponad*).mp. | 178 |
| 4 | (pericardia* adj2 tamponad*).mp. | 69 |
| 5 | pericardial effusion/ | 88 |
| 6 | (pericardial adj2 effus*).mp. | 659 |
| 7 | (peri-cardial adj2 effus*).mp. | 0 |
| 8 | (pericardi* adj2 tamponad*).mp. | 91 |
| 9 | (peri-cardi* adj2 tamponad*).mp. | 0 |
| 10 | (heart? adj2 tamponad*).mp. | 257 |
| 11 | (myocardia* adj2 tamponad*).mp. | 3 |
| 12 | chylopericardi???.mp. | 0 |
| 13 | haemopericardi???.mp. | 4 |
| 14 | hemopericardi???.mp. | 45 |
| 15 | ((compress* adj3 heart?) and (increas* adj3 intrapericardial pressure?)).mp. | 0 |
| 16 | ((compress* adj3 heart?) and (increas* adj3 intra-pericardial pressure?)).mp. | 0 |
| 17 | pericarditis/ | 32 |
| 18 | or/1-17 [ Cardiac Tamponade & related terms ] | 1001 |
| 19 | Appendicitis/ | 502 |
| 20 | Appendix/ | 31 |
| 21 | appendectomy/ | 466 |
| 22 | exp appendix disease/ [ Embase ] | 0 |
| 23 | appendicit*.mp. | 1426 |
| 24 | appendix??.mp. | 2187 |
| 25 | appendices.mp. | 109 |
| 26 | appendiceal*.mp. | 145 |
| 27 | appendicular*.mp. | 452 |
| 28 | appendicolith*.mp. | 11 |
| 29 | appendicectom*.mp. | 336 |
| 30 | appendectom*.mp. | 1250 |
| 31 | appendicopath*.mp. | 1 |
| 32 | caecal appendage?.mp. | 0 |
| 33 | cecal appendage?.mp. | 0 |
| 34 | vermiform process??.mp. | 0 |
| 35 | vermix.mp. | 0 |
| 36 | or/19-35 [ related to Appendix ] | 4363 |
| 37 | 18 and 36 [ Cardiac Tamponade + Appendix ] | 7 |
| 38 | conferenc*.so. | 42564 |
| 39 | confere*.pt. | 17083 |
| 40 | (book or book article or book book or book note or "book review" or book series article or book series article in press or book series chapter or book series conference paper or book series letter or "book series review" or book series short survey or chapter or conference abstract or conference abstract placebo controlled partly blinded crossover study in 12 sle patients or conference proceeding or "conference review" or journal conference abstract or "journal conference review").pt. | 164718 |
| 41 | 38 or 39 or 40 | 166788 |
| 42 | 37 not 41 | 5 |
| 43 | remove duplicates from 42 | 5 |

# CDSR

Cochrane Database of Systematic Reviews 2005 to Present

| **#** | **Searches** | **Results** |
| --- | --- | --- |
| 1 | (cardia? adj2 tamponad*).ti,ab. | 2 |
| 2 | (pericardia* adj2 tamponad*).ti,ab. | 0 |
| 3 | (pericardial adj2 effus*).ti,ab. | 1 |
| 4 | (peri-cardial adj2 effus*).ti,ab. | 0 |
| 5 | (pericardi* adj2 tamponad*).ti,ab. | 0 |
| 6 | (peri-cardi* adj2 tamponad*).ti,ab. | 0 |
| 7 | (heart? adj2 tamponad*).ti,ab. | 0 |
| 8 | (myocardia* adj2 tamponad*).ti,ab. | 0 |
| 9 | chylopericardi???.ti,ab. | 0 |
| 10 | haemopericardi???.ti,ab. | 0 |
| 11 | hemopericardi???.ti,ab. | 0 |
| 12 | ((compress* adj3 heart?) and (increas* adj3 intrapericardial pressure?)).ti,ab. | 0 |
| 13 | ((compress* adj3 heart?) and (increas* adj3 intra-pericardial pressure?)).ti,ab. | 0 |
| 14 | pericarditis.ti,ab. | 2 |
| 15 | or/1-14 [ Cardiac Tamponade & related terms ] | 4 |
| 16 | appendicit*.ti,ab. | 13 |
| 17 | appendix??.ti,ab. | 7 |
| 18 | appendices.ti,ab. | 1 |
| 19 | appendiceal*.ti,ab. | 1 |
| 20 | appendicular*.ti,ab. | 0 |
| 21 | appendicolith*.ti,ab. | 0 |
| 22 | appendicectom*.ti,ab. | 5 |
| 23 | appendectom*.ti,ab. | 7 |
| 24 | appendicopath*.ti,ab. | 0 |
| 25 | caecal appendage?.ti,ab. | 0 |
| 26 | cecal appendage?.ti,ab. | 0 |
| 27 | vermiform process??.ti,ab. | 0 |
| 28 | vermix.ti,ab. | 0 |
| 29 | or/16-28 [ Appendix & related terms ] | 17 |
| 30 | 15 and 29 [ Cardiac Tamponade + Appendix ] | 0 |
